# A universal monoclonal antibody protects against all influenza A and B viruses by targeting a highly conserved epitope in the viral neuraminidase

**DOI:** 10.1186/1471-2164-15-S2-P8

**Published:** 2014-04-02

**Authors:** Tracey M Doyle, Anwar M Hashem, Changgui Li, Doris J Bucher, Gary Van Domselaar, Junzhi Wang, Terry Cyr, Aaron Farnsworth, Runtao He, Aeron C Hurt, Earl G Brown, Xuguang Li

**Affiliations:** 1 Centre for Vaccine Evaluation, Biologics and Genetic Therapies Directorate, HPFB, Health Canada, Ottawa, ON, Canada; 2 Department of Biochemistry, Microbiology and Immunology, University of Ottawa, Ottawa, ON, Canada; 3 Department of Medical Microbiology and Parasitology, Faculty of Medicine, King Abdulaziz University, Jeddah, Saudi Arabia; 4 Special Infectious Agents Unit, King Fahd Medical Research Centre, King Abdulaziz University, Jeddah, Saudi Arabia; 5 National Institutes for Food and Drug Control, Beijing, China; 6 Department of Microbiology & Immunology, New York Medical College, Valhalla, NY 10595, USA; 7 National Microbiology Laboratory, Public Health Agency of Canada, Winnipeg, MB, Canada; 8 WHO Collaborating Centre for Reference and Research on Influenza, 10 Wreckyn St., North Melbourne, Victoria 3051, Australia; 9 Emerging Pathogens Research Centre, University of Ottawa, Ottawa, ON, Canada

## Background

Hemagglutinin (HA) and neuraminidase (NA) are the two major surface glycoproteins of influenza viruses and the main targets of vaccine-induced antibodies (Abs). While several broadly neutralizing Abs targeting conserved epitopes in diverse HA subtypes have been isolated, NA-specific Abs could only cross-protect partially against homologous and heterologous strains from the same subtype.

## Materials and methods

Comprehensive bioinformatics analyses of all publicly available full-length NA sequences using multiple alignments and Shannon entropy were conducted to identify conserved sequences in all influenza A and B viral NA [1]. Growth kinetics of wild-type or recombinant viruses with single alanine substitutions within the identified regions was then analyzed in MDCK cells. A rabbit monoclonal Ab (mAb), denoted as HCA-2, raised against one of the characterized sequences was then examined for its in vitro inhibitory effects and in vivo prophylactic efficacy against several influenza A and B strains.

## Results

Bioinformatics analyses uncovered a universally conserved 9-mer peptide amongst all influenza NA proteins (amino acids 222-230 and comprised of “ILRTQESEC”). Substitutions within this universal epitope underscored its crucial roles in viral fitness and replication [2]. Importantly, the HCA-2 mAb showed broad in vitro inhibition against multiple strains from all influenza A NA subtypes (N1-N9) and influenza B viruses from both Victoria and Yamagata genetic lineages [3,4]. It also provided heterosubtypic protection in mice against lethal doses of H1N1 and H3N2 strains. Finally, amino acid residues I222 and E227, located in close proximity to the active site, were found to be indispensable for inhibition by this mAb [3,4].

## Conclusions

These findings reveal the essential role of this unique highly-conserved sequence in NA function and viral replication and indicate that it is sufficiently exposed to allow access by inhibitory antibody during the course of infection. Thus, it could represent a potential target for novel antivirals or targeted-vaccines against diverse strains of influenza A and B viruses.
